# Correlation of dsm-5-based and hads self-reported depression phenotypes: Preliminary results of on-line survey in russian population cohort

**DOI:** 10.1192/j.eurpsy.2021.892

**Published:** 2021-08-13

**Authors:** G. Rukavishnikov, A. Rakitko, E. Kasyanov, V. Ilinsky, A. Kibitov, G. Mazo, N. Neznanov

**Affiliations:** 1 Department Of Translational Psychiatry, V. M. Bekhterev National Medical Research Center for Psychiatry and Neurology, St. Petersburg, Russian Federation; 2 N/a, Genotek Ltd., Moscow, Russian Federation; 3 Department Of Geriatric Psychiatry, Bekhterev National Medical Research Center for Psychiatry and Neurology, St. Petersburg, Russian Federation; 4 V.P. Serbsky National Medical Research Center on Psychiatry and Addictions, Russia

**Keywords:** Depression, phenotyping, screening scales, DSM criteria

## Abstract

**Introduction:**

To reduce the heterogeneity of depressive GWAS samples it seems relevant to evaluate and compare current instruments for depression phenotyping.

**Objectives:**

The aim is to evaluate the agreement of DSM criteria and HADS scores in depression phenotyping for population studies.

**Methods:**

The self-report data was obtained from 5116 clients (females 50,63%; mean age 36,92±9,82 years, Ме=42; Q1=35, Q3=76) of genetic testing company Genotek Ltd.. The respondents completed an on-line questionnaire with items on social status and biometrics. Depression phenotyping was based on DSM-5 criteria (life-time and current for major and bipolar depression) and HADS (current).

**Results:**

Mean HADS scores were: HADS-A – 6,43±2,9, Ме=8; Q1=6, Q3=18; HADS-D – 4,5±2,83, Ме=6; Q1=4, Q3=17. Abnormal anxiety and depression (≥11 for each subscale) were present in 9% (N–456) and 3,4% (N–174) of respondents, respectively; borderline (8-10) – in 23% (N–1172) and 11,9% (N–592), respectively. The life-time report of major depression according to DSM-5 criteria was 17,6% (N–261) and of bipolar disorder – 8,3% (N–139). Moderate correlations were present for borderline HADS anxiety scores and DSM major depression (0.19, p<.01). Similar correlations of HADS anxiety scores were registered for DSM bipolar depression (0.20, p<.01). Moreover, HADS depression scores did not correlate with any DSM depressive phenotype.

**Conclusions:**

Our study shows significant correlations only for DSM depression criteria and HADS anxiety, but not depression scores. It could indicate the different significance of individual scale items in depression phenotyping and the need for their separate further evaluation.

**Conflict of interest:**

The research is supported by the Russian Scientific Fund grant #20-15-00132.

